# Supplementary material for: Custom-Made 3D-Printed Implants for Anterior Column Reconstruction in the Upper Cervical Spine after Intralesional Extracapsular Excision—Report of 2 Cases and Literature Review
Source: J Clin Med. 2022 Oct 13;11(20):6058. doi: 10.3390/jcm11206058 (PMC9604658; doi:10.3390/jcm11206058)
Supplement: Supplementary file 1 [file jcm-11-06058-s001.zip › jcm-1909574-supplementary.pdf]

itc INSTITUTO TECNOLÓGICO DE CANARIAS

Gobierno de Canarias

osteobionix

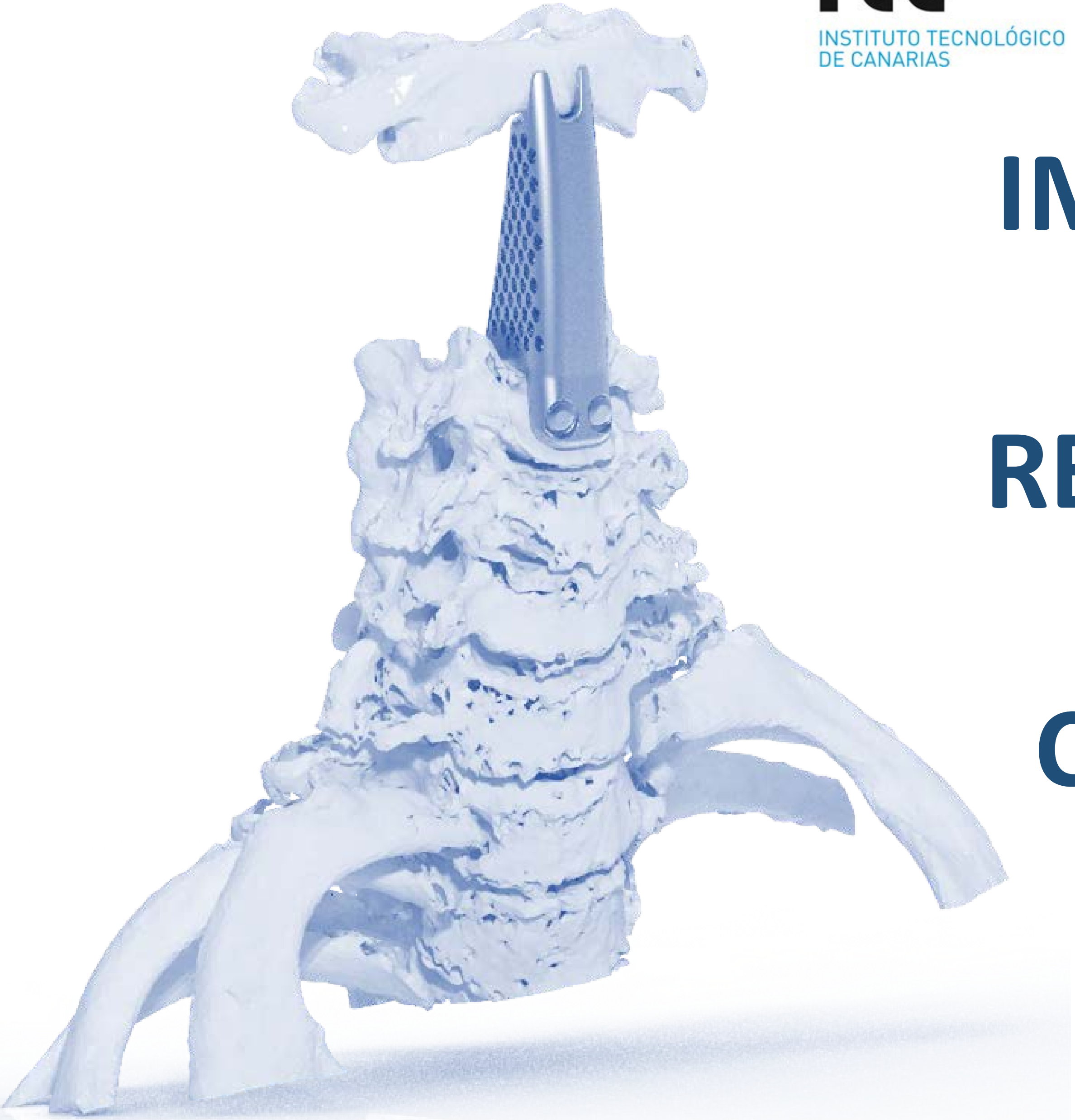

IMPLANTE A MEDIDA PARA LA RECONSTRUCCIÓN DE LAS VÉRTEBRAS CERVICALES C2 Y C3

IMPLANTE CERVICAL: RECONSTRUCCIÓN ANATÓMICA

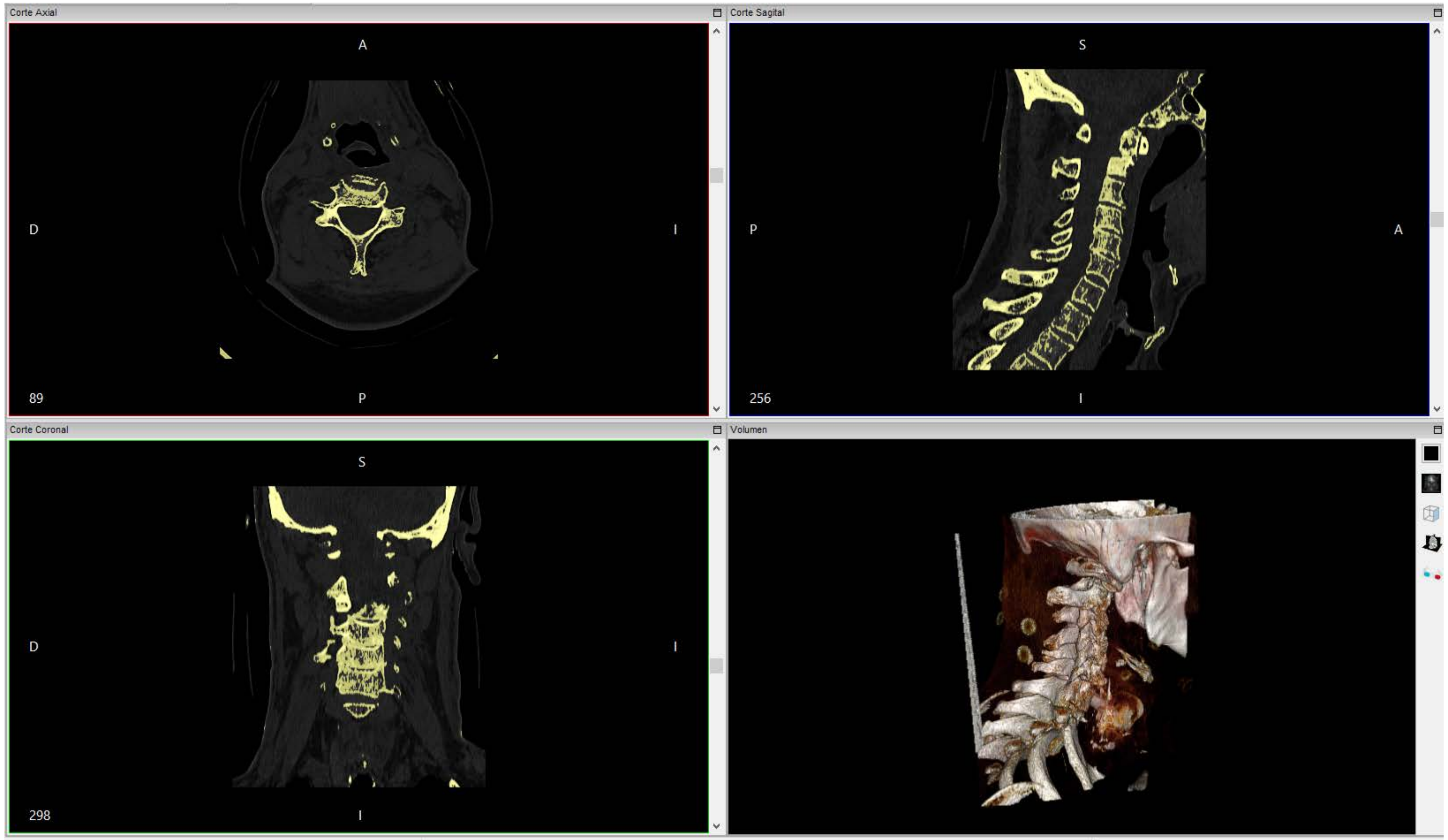

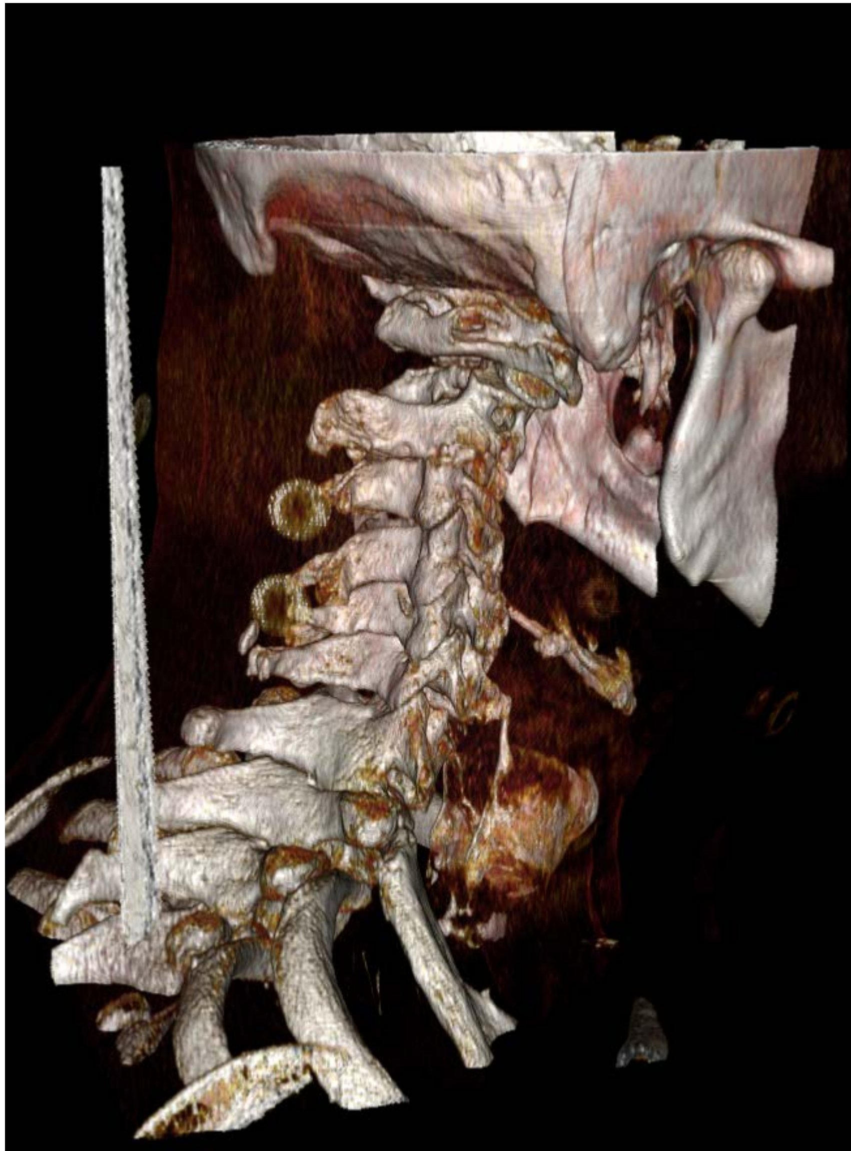

IMPLANTE PARA EL SACRO: RECONSTRUCCIÓN ANATÓMICA

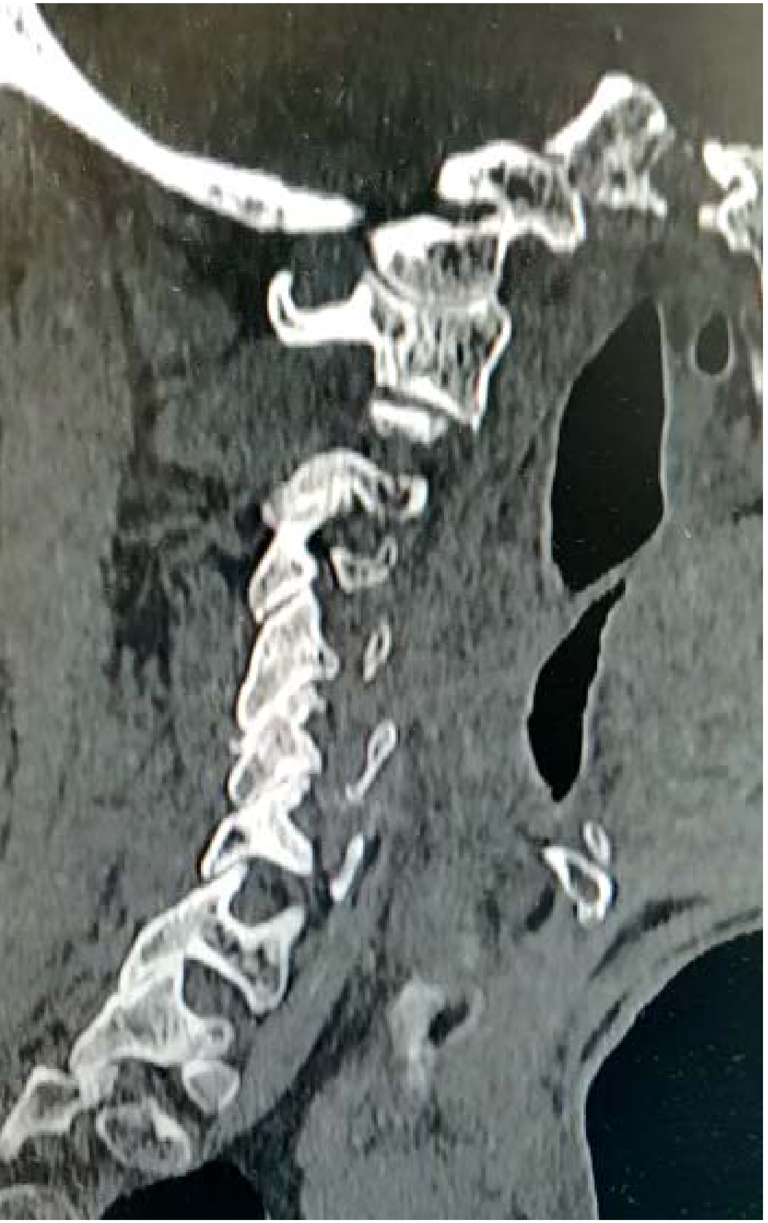

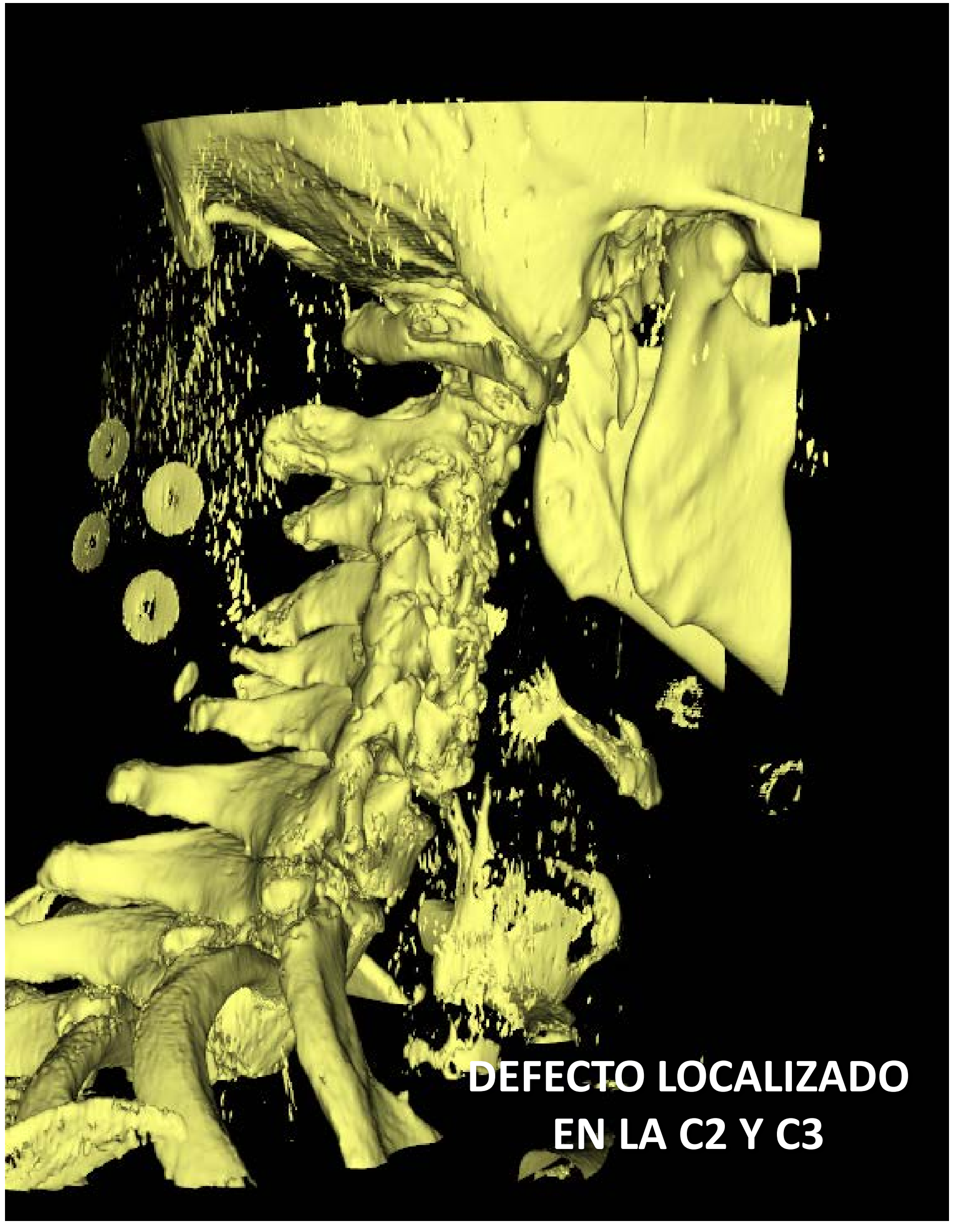

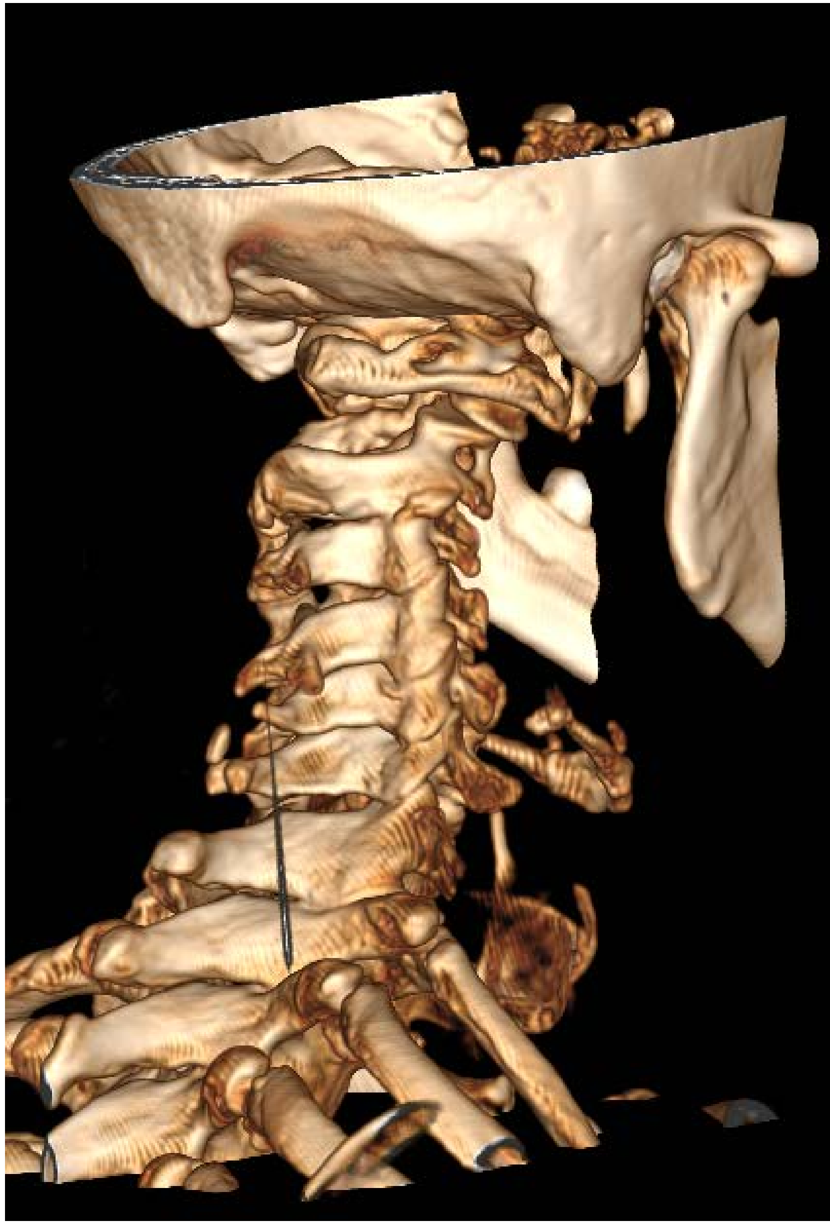

IMPLANTE CERVICAL: PREPARACIÓN DE MODELOS

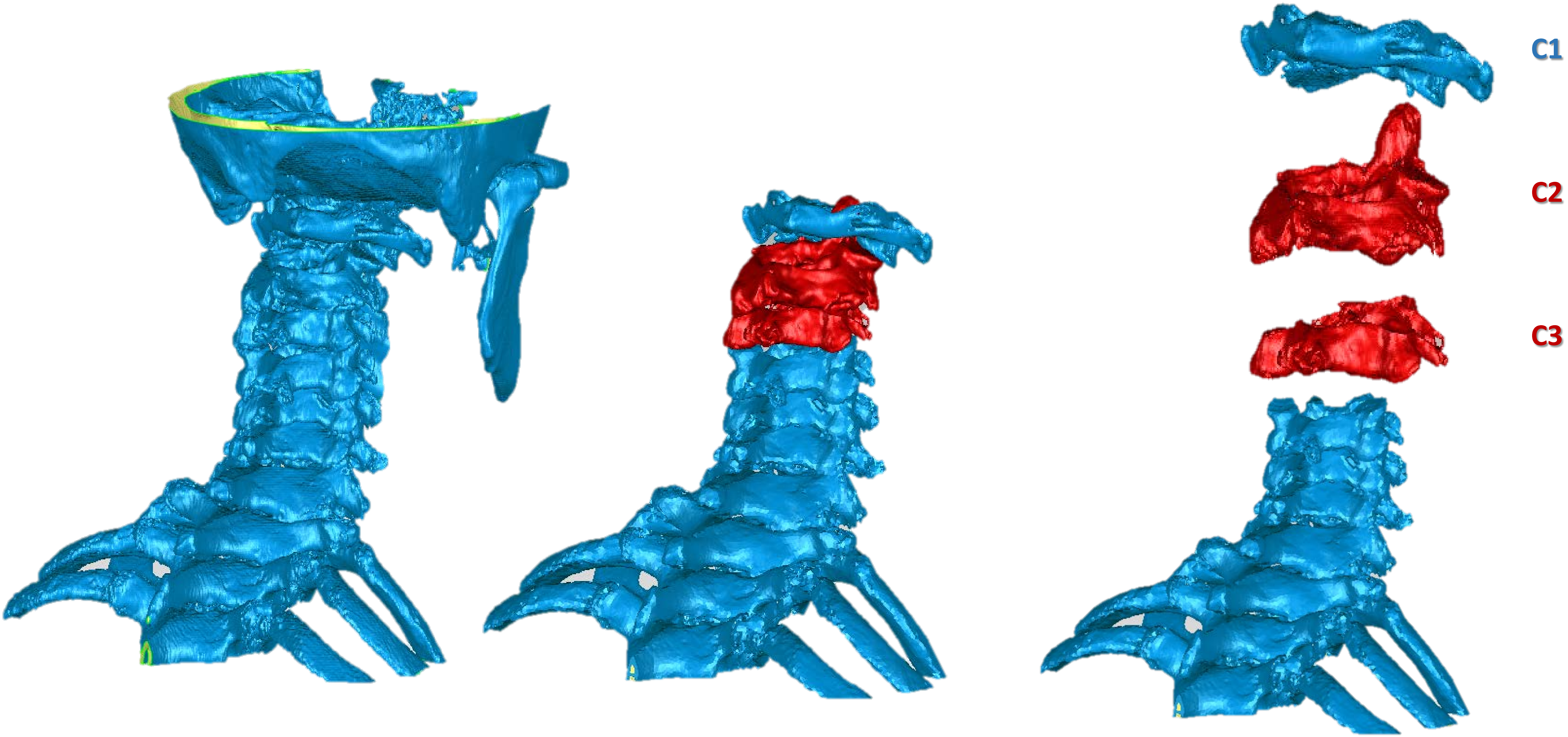

IMPLANTE CERVICAL: PLANIFICACIÓN

MODELO ANATÓMICO

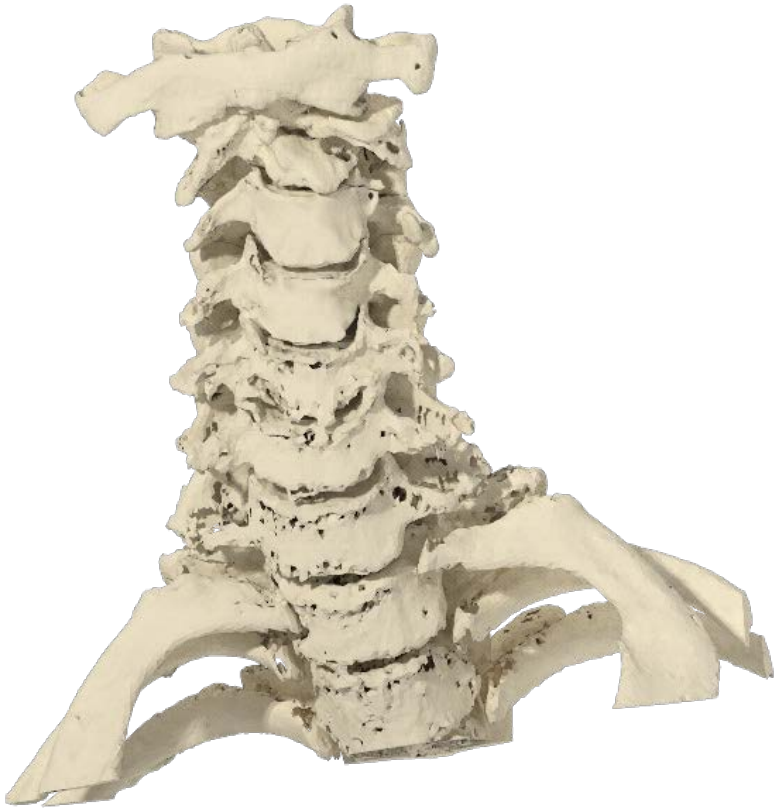

REUBICACIÓN DE LA C1

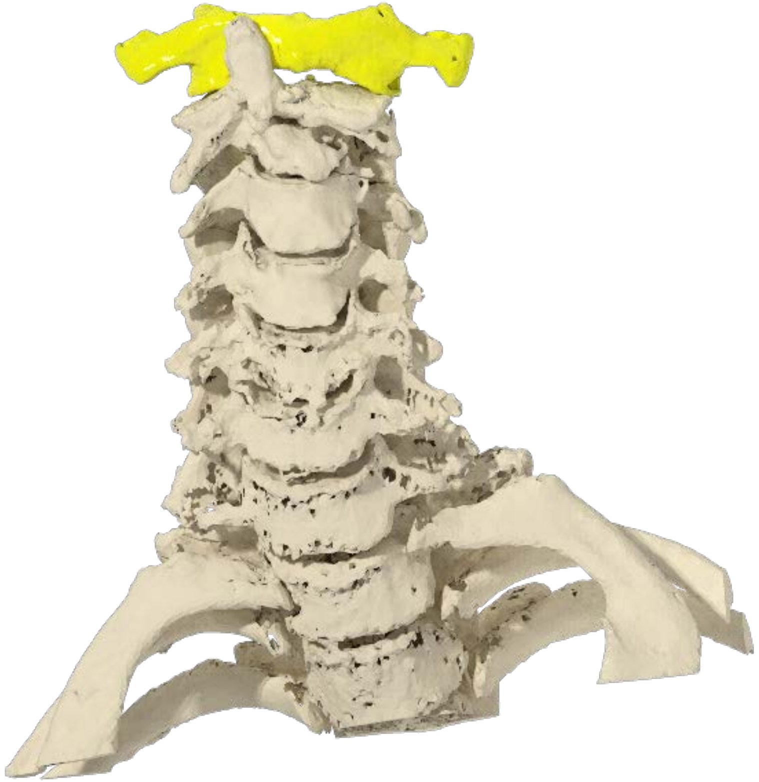

MODELO DE TRABAJO MÁS DEFECTO

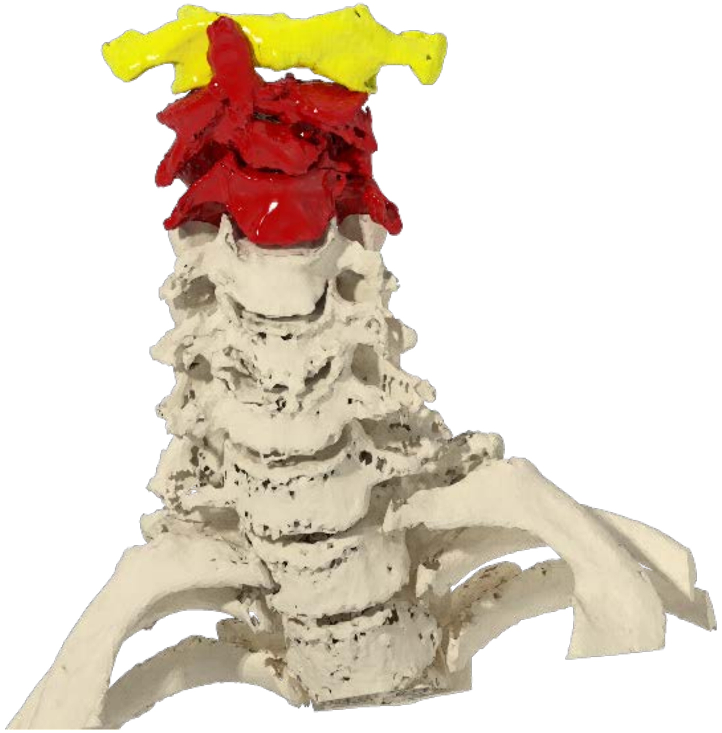

ABORDAJE ANTERIOR

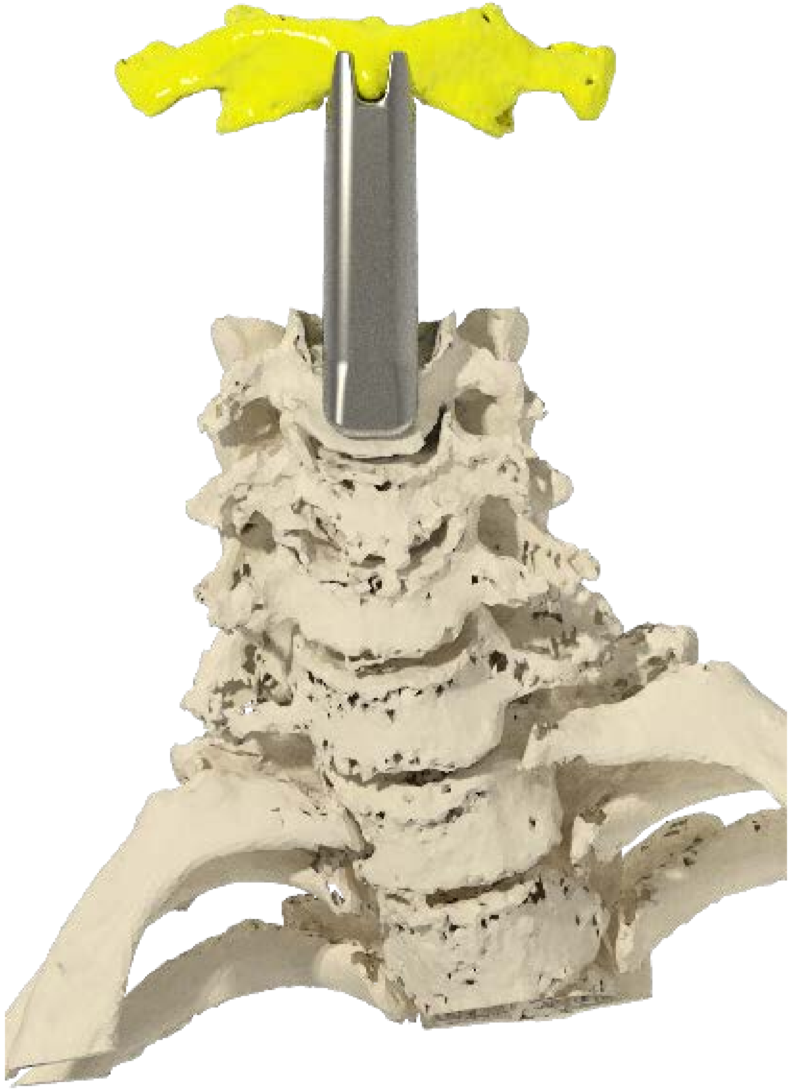

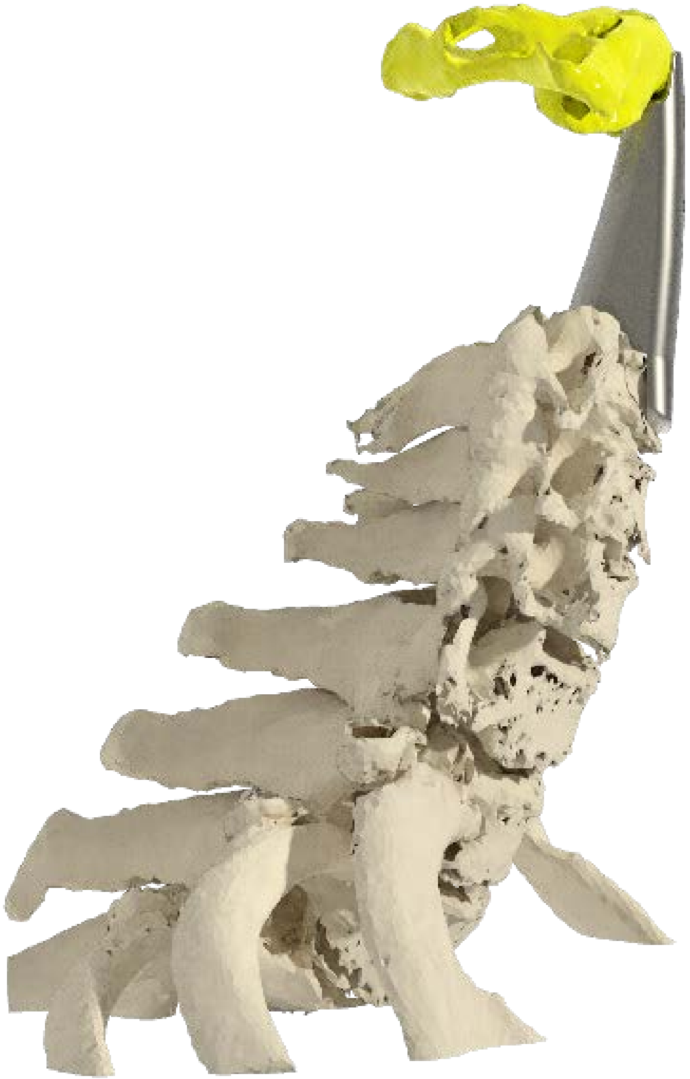

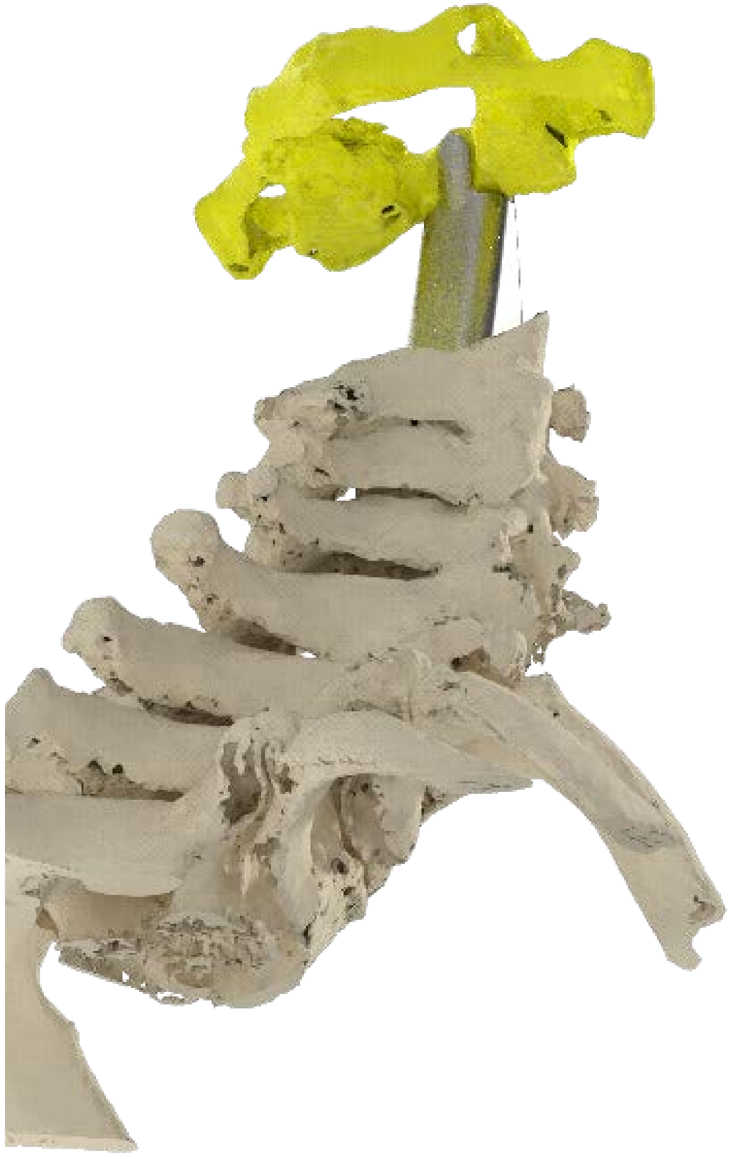

IMPLANTE CERVICAL: DISEÑO DE IMPLANTE

ABORDAJE ANTERIOR

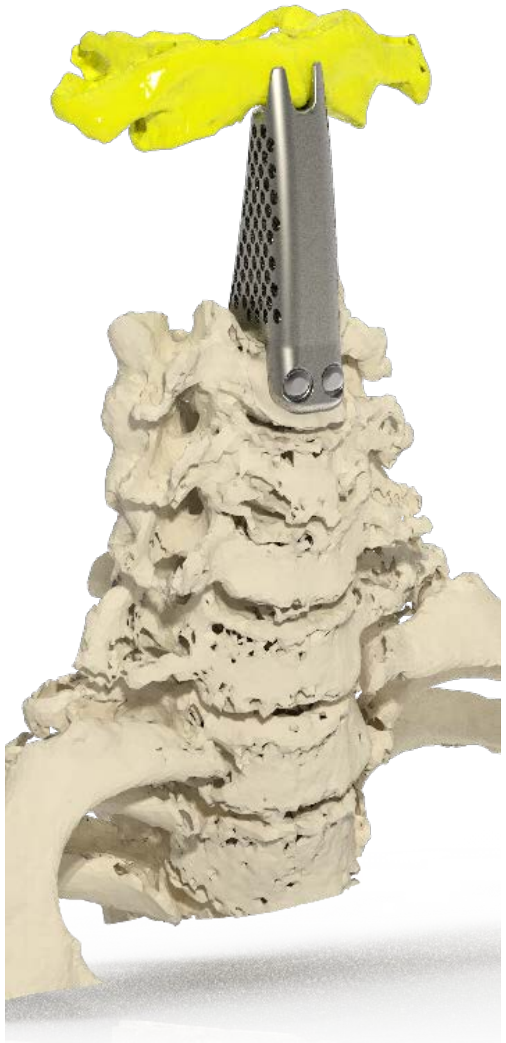

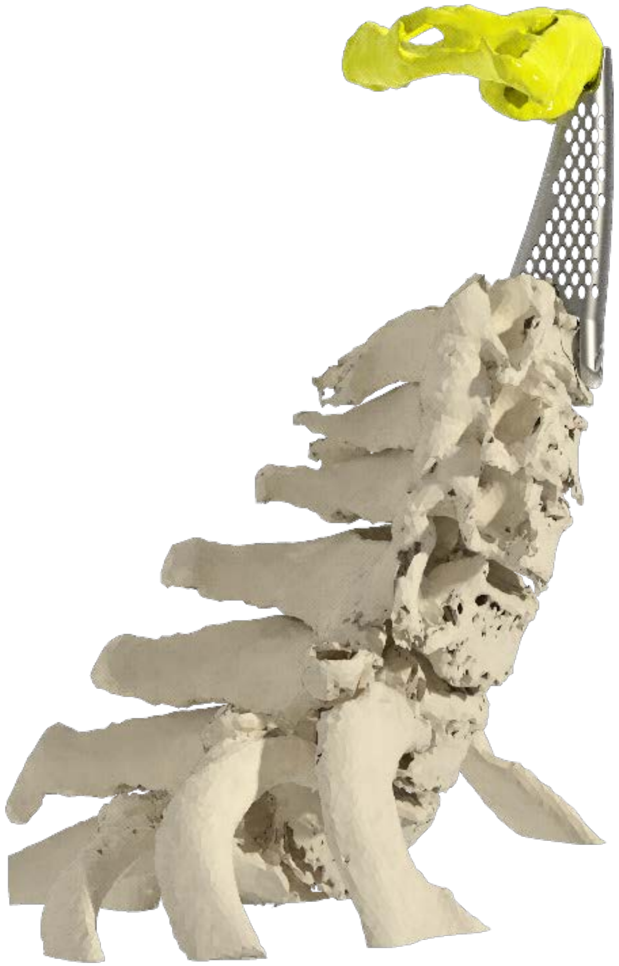

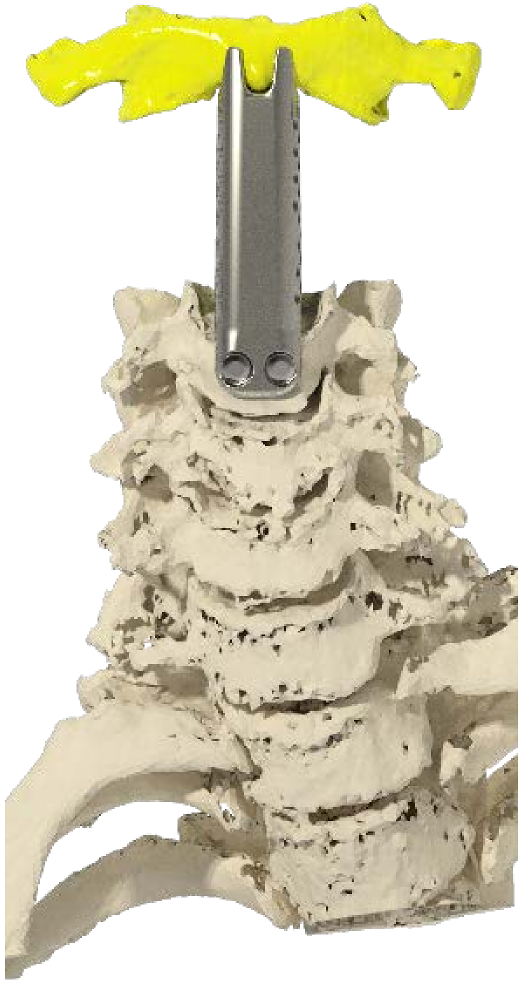

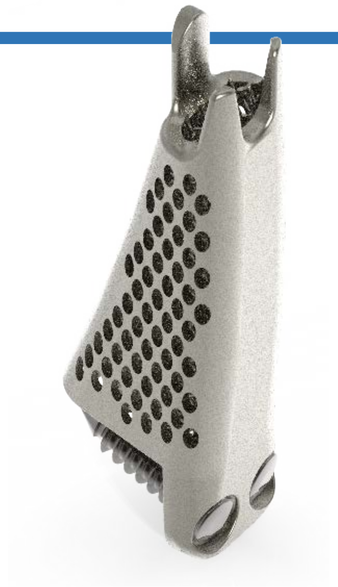

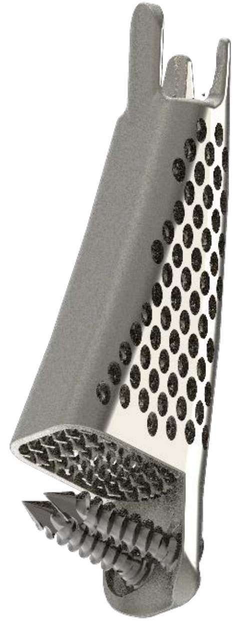

IMPLANTE CERVICAL: DISEÑO DE IMPLANTE

DESFASE DE 2MM
ENTRE CADA IMPLANTE

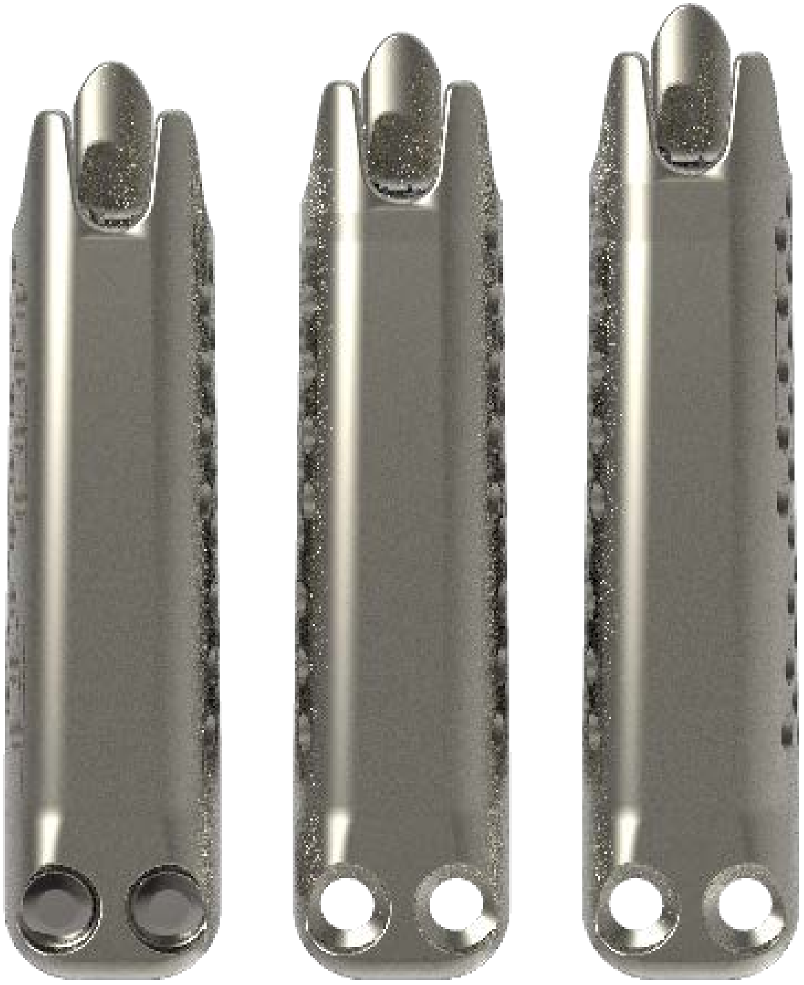

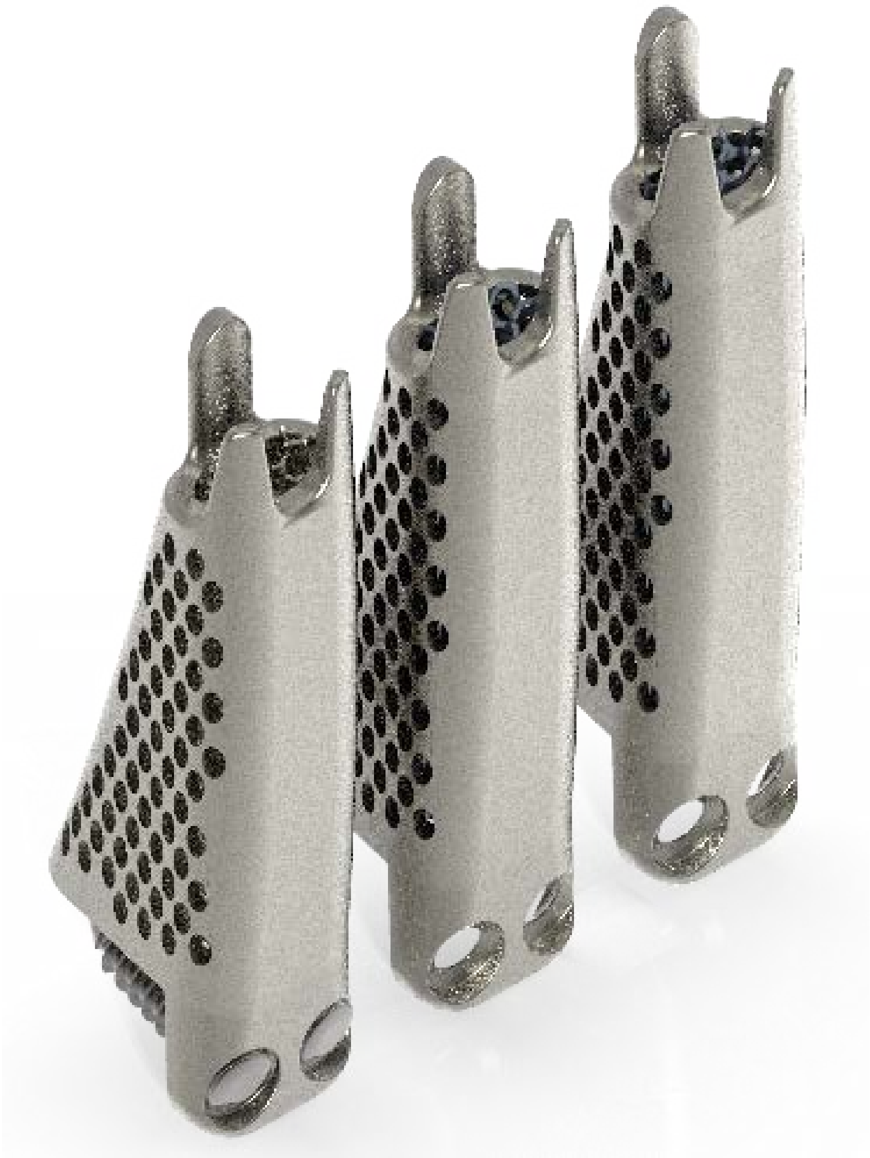

IMPLANTE CERVICAL: FABRICACIÓN

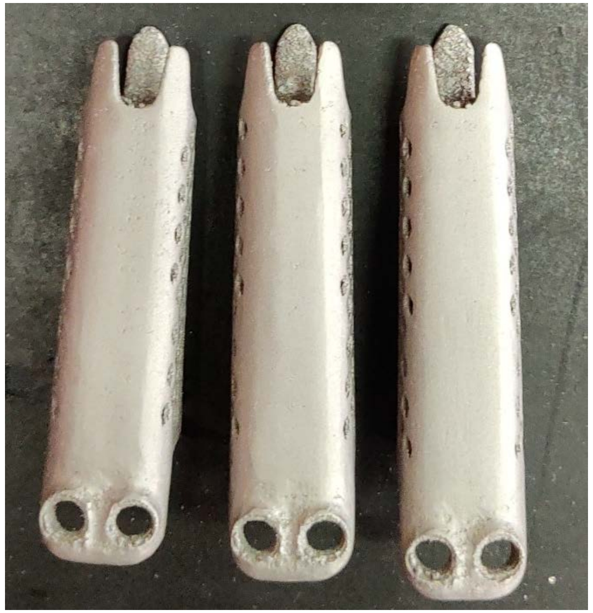

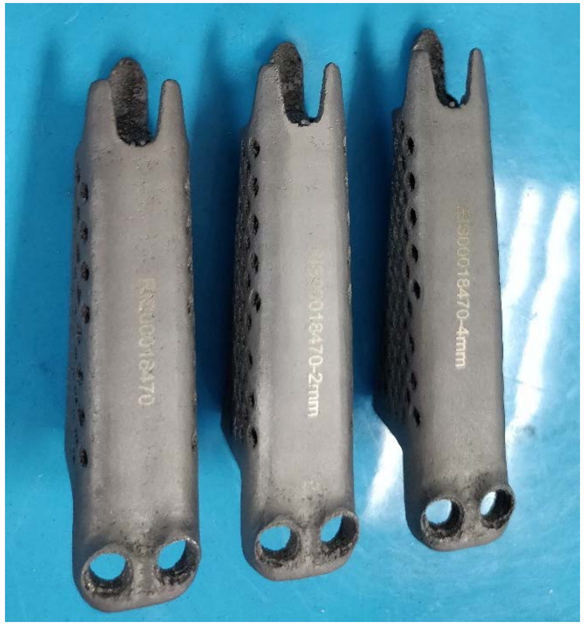

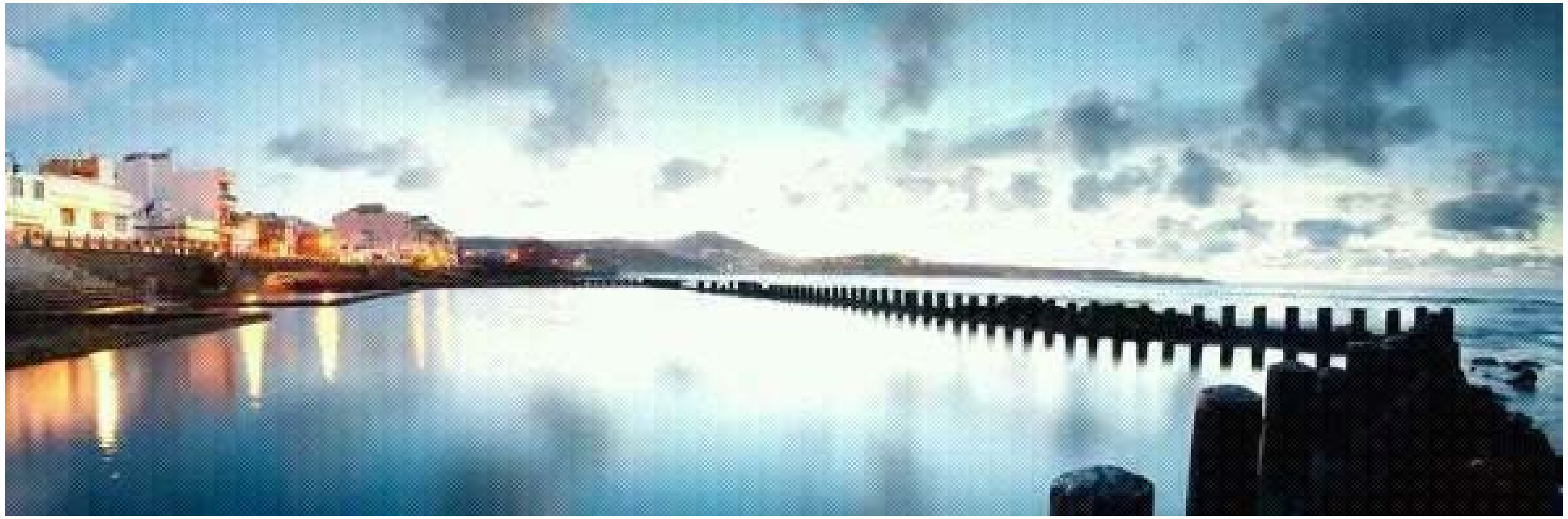

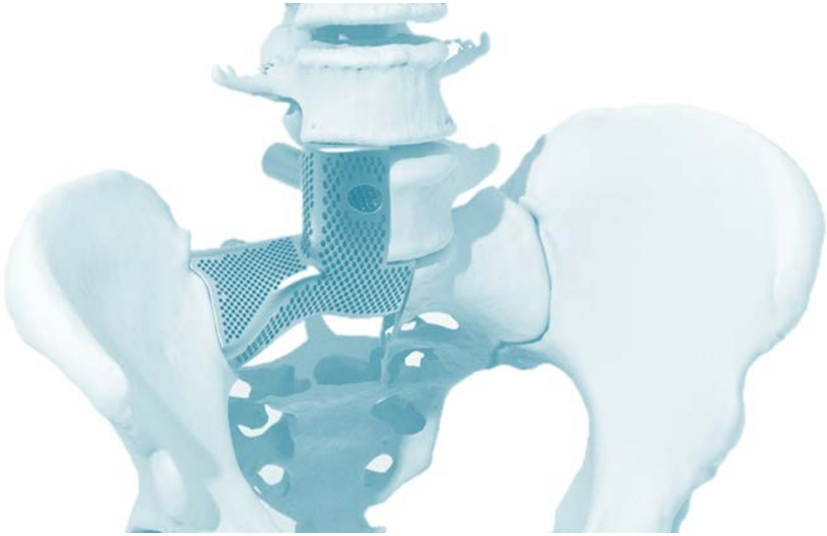

Muchas Gracias

INSTITUTO TECNOLÓGICO DE CANARIAS

Gobierno de Canarias

Donato Monopoli
Ingeniero biomédico
dmonopoli@itccanarias.org

osteobionix
